# affy2sv: an R package to pre-process Affymetrix CytoScan HD and 750K arrays for SNP, CNV, inversion and mosaicism calling

**DOI:** 10.1186/s12859-015-0608-y

**Published:** 2015-05-20

**Authors:** Carles Hernandez-Ferrer, Ines Quintela Garcia, Katharina Danielski, Ángel Carracedo, Luis A. Pérez-Jurado, Juan R. González

**Affiliations:** 1 Center for Research in Environmental Epidemiology (CREAL), Doctor Aiguader 88, 08003 Barcelona, Spain; 2 Universitat Pompeu Fabra (UPF), Barcelona, Spain; 3 CIBER Epidemiología y Salud Pública (CIBERESP), Madrid, Spain; 4 Grupo de Medicina Xenómica - Universidade de Santiago de Compostela, Santiago de Compostela, Spain; 5 Centro Nacional de Genotipado - Instituto Carlos III, Santiago de Compostela, Spain; 6 Affymetrix, UK Ltd, High Wycombe, UK; 7 CIBER Enfermedades Raras (CIBERER), Madrid, Spain; 8 Fundación Pública Galega de Medicina Xenómica (SERGAS), Santiago de Compostela, Spain; 9 King Abdulaziz University, Center of Excellence in Genomic Medicine Research, Jeddah, Saudi Arabia; 10 Departament de Ciències Experimentals i de la Salut, Universitat Pompeu Fabra (UPF), Barcelona, Spain; 11 IMIM (Hospital del Mar Medical Research Institute), Barcelona, Spain

**Keywords:** Affymetrix, CytoScan, CytoScan HD, CytoScan 750k, CNV, Inversion, Mosaicism, Structural variants

## Abstract

**Background:**

The well-known Genome-Wide Association Studies (GWAS) had led to many scientific discoveries using SNP data. Even so, they were not able to explain the full heritability of complex diseases. Now, other structural variants like copy number variants or DNA inversions, either germ-line or in mosaicism events, are being studies. We present the R package affy2sv to pre-process Affymetrix CytoScan HD/750k array (also for Genome-Wide SNP 5.0/6.0 and Axiom) in structural variant studies.

**Results:**

We illustrate the capabilities of affy2sv using two different complete pipelines on real data. The first one performing a GWAS and a mosaic alterations detection study, and the other detecting CNVs and performing an inversion calling.

**Conclusion:**

Both examples presented in the article show up how affy2sv can be used as part of more complex pipelines aimed to analyze Affymetrix SNP arrays data in genetic association studies, where different types of structural variants are considered.

**Electronic supplementary material:**

The online version of this article (doi:10.1186/s12859-015-0608-y) contains supplementary material, which is available to authorized users.

## Background

Genome-Wide Association Studies (GWAS) interrogate a large number of genetic variants with high-throughput technologies using single nucleotide polymorphisms (SNPs). Up to now, GWAS have led to many scientific discoveries including genes and gene variants related to cancer [1–4], asthma [5–7] or obesity [8, 9] among others. Nonetheless, SNPs have explained relatively little of the total heritability of complex diseases [10, 11]. In order to overcome this difficulty, researchers are also analyzing other structural genomic variants (SVs) such as copy number variants (CNVs) [12–14], inversions [15, 16] or chromosomal rearrangements present in mosaicism [17–19]. This has been possible due to the efforts made by scientific community in developing new tools to detect SV using existing SNP array data [20–22].

Over the last few years, commercial enterprises such as Affymetrix and Illumina, have produced high-density SNP arrays that made possible to genotype many markers in a single assay. These arrays are excellent tools to perform GWAS not only with SNPs but also with common and rare SVs. An example of it is Affymetrix CytoScan family, that includes a high-density array (CytoScan HD) and a light version array (CytoScan 750K) [23, 24]. This family of arrays was designed to provide a genome-wide overview of the whole genome since they include markers for constitutional and cancer genes and *OMIM* and *RefSeq* genes.

Affymetrix provides a wide range of software to analyze the data obtained from their arrays. The most common software to analyze CytoScan data is called Chromosome Analysis Suite (ChAS) [25]. Despite the benefits, the usage of ad hoc software from Affymetrix has two main limitations. On one hand, while the raw data can be processed in a high throughput way, the analysis of the results is recommended to be performed by groups of three subjects. On the other hand, the set of available analysis is reduced to the algorithms included in the software, so no other custom-functionality can be added to help researchers to perform downstream analyses.

In order to overcome these drawbacks an R package called affy2sv has been created. This R package improves the advantages provided by ChAS incorporating new functionalities that make possible the analysis of CytoScan data using other existing R packages (MAD [26], R-GADA [27], snpStats [28], invClust [29, 30]) and external software (PLINK [31], PennCNV [32–34]), as well as data visualization. Therefore, affy2sv will facilitate the analysis of CytoScan data in SNPs, CNVs, mosaicism or inversion association studies using pipelines under R environment.

In this article, we illustrate affy2sv's performance by analyzing two different sets of SNP array generated with CytoScan platform. The first set includes population of two different locations: 429 subjects from general population of Toronto and 198 subjects from Nijmegen (Dataset A). The second set includes 315 subjects diagnosed with intellectual disability (Dataset B). Dataset A is used to illustrate how to compare genetic variants between two general populations under GWAS framework and how to detect mosaicism events. Dataset B is used to illustrate how to detect potentially pathogenic CNVs and how to perform inversion calling. The result obtained from the inversion analysis is the genotype of a well-known inversion located at chromosome 8p23.1 [35].

## Implementation

affy2sv is implemented as a R package freely available from its web page [36] and through CREAL-installer [37]. affy2sv is based on standard CRAN and Bioconductor classes allowing for full flexibility, modularity and integration with other R packages.

### Input data

affy2sv is compatible with the newest Affymetrix SNP array CytoScan HD/750k, but it also accepts Genome-Wide SNP 5.0/6.0 and Axiom arrays. It works with the raw data files, known as .CEL files. Internally, affy2sv uses the package CRLMM [38–41] to extract some measures [genotype, Log R Ratio (LRR) and B Allele Frequency (BAF)] from Genome-Wide SNP 5.0/6.0 raw data. To deal with Axiom and CytoScan arrays and to extract the homologous measures (genotype, allele peaks, allele intensities, LRR and BAF), affy2sv uses the Affymetrix Power Tools (APT) [42].

### Output data

affy2sv can be used to process .CEL files and to generate R objects and files compatibles with snpStats, MAD, R-GADA, PLINK, and PennCNV. These R packages and programs are specifically designed to perform GWAS, analyze mosaicism and CNVs, respectively.

The R object generated for snpStats is called *SnpMatrix Container*. This object contains a MAP and a SnpMatrix. The MAP is a data.frame that includes an annotation for each SNP (SNP's name, chromosome, cM, position and alleles). The genotypes are stored in a SnpMatrix object. The file compatible with MAD and R-GADA is a tabular file for each subject containing the BAF, the LRR and the genotype of each SNP (SNP's name, chromosome, position, LRR, BAF and genotype). The compatibility with PLINK is reached creating a TPED file (transposed format), which contains the chromosome, SNP's name, genetic distance and position, followed by all the genotype-pairs. To work with PennCNV several files are required. The tools manual, available on its web page [43], explains its composition and how to generate them. affy2sv creates the a file that contains the LRR, BAF and genotype, called signal intensity file.

## Method

**Fig. 1:**
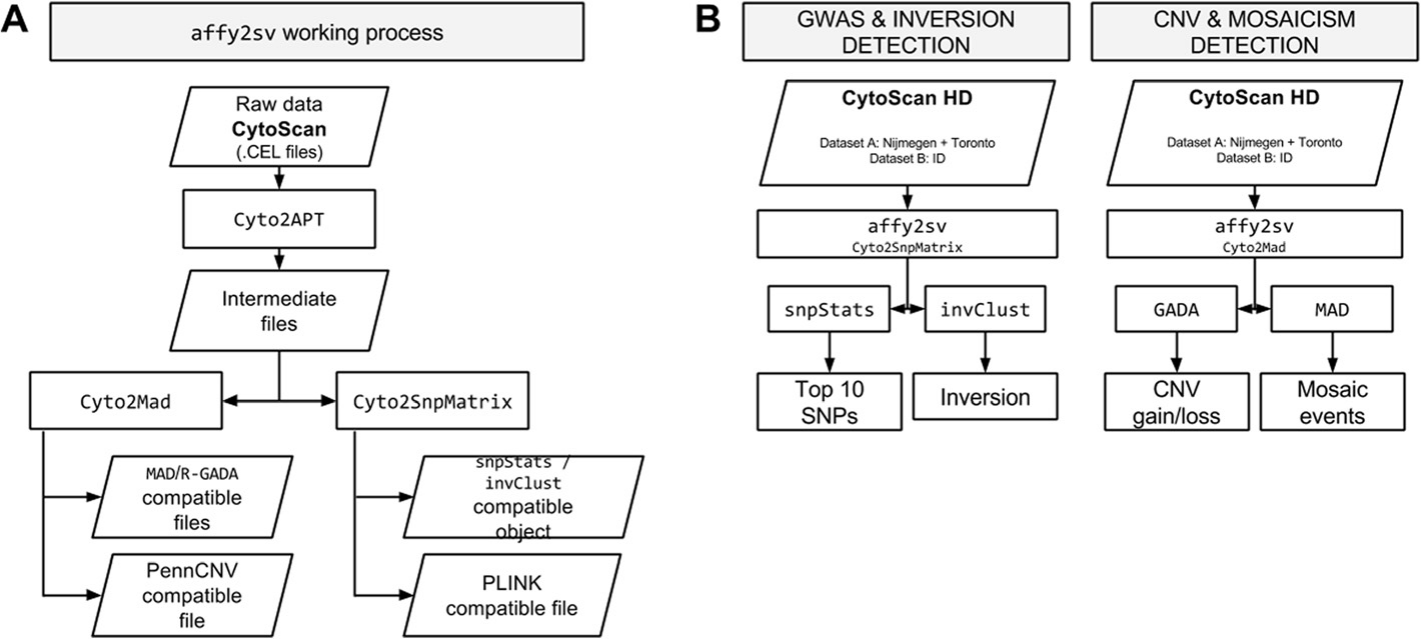
Schema of the application of affy2sv to analyze CytoScan data. Part **A** of the figure shows the work-flows available in the R package affy2sv. These work-flows are composed by two steps: generate intermediate files and generate specific output. First step us done using the function Cyto2APT. The seconds step is done using the functions Cyto2Mad and Cyto2SnpMatrix. Part **B** of the figure shows the pipelines used to perform the two studies detailed in the article. The two CytoScan HD populations were pre-process using affy2sv and then analyzed using different tools

affy2sv is a set of R functions used to process a certain type of raw data and generate a specific output file. There are two steps to process the data from Affymetrix CytoScan arrays with affy2sv: 1) read raw data and calculate measures (genotype, LRR and BAF) 2) generate a specific output. This two-step process is illustrated in Fig. 1a. The figure shows that *intermediate files* are created with the first step. Also that these *intermediate files* are used as input for the second step and used to generate a specific output.

### Step 1: Process raw data and get BAF, LRR and genotype

This step is performed using the function Cyto2APT. Cyto2APT is in charge to call the APT. These tools require a series of library and annotation files depending on the array-technology used. These files can be downloaded from the Affymetrix Library [44] and from the Affymetrix annotation [45] web pages. The user needs to download the files corresponding to their own data's technology. Later, the function APTparam creates a required object that indicates the correct system call to deal with apt-copynumber-cyto from APT. The following code illustrates the use of a standard call:

This code indicates that the raw .CEL files are located at /home/cydata. The argument output.path indicates where the *intermediate files* will be saved. In analysis.path is indicated the path where all the library and annotation files are stored. All the other arguments refer to the library and annotation files required by the function. These argument define the technology used in the array, the distribution of the probes, the name of each probe (and the related SNP) and others.

We thought these technical arguments could be hidden, but leaving them unmasked would allow the user to have more than one library (for example, one for CytoScan HD and another one for CytoScan 750K) or more than one version of a single library. The term *intermediate files* is used to refer to the files generated by Cyto2APT. These files are, in fact, the plain text version of the common .cychp files generated by apt-copynumber-cyto. So, at the end of this step, the intermediate files generated by Cyto2APT are the same files that could be obtained by using ChAS. This is because the system call to apt-copynumber-cyto generated by affy2sv is the recommended by Affymetrix in the tool's manual [46, 47].

In order to increase the versatility of the package affy2sv, we also make possible to create a personalized system call to apt-copynumber-cyto through APTparam. This can be done by setting the argument type from standard to custom . Then, it is needed to fill the argument param with a string containing all the arguments for apt-copynumber-cyto (arguments like cel.list, output.path… must not to be set on APTparam but in the string to param). An example of how to do it is available in the supplementary material (Additional file 1).

Once APTparam set up the arguments, Cyto2APT will manage with apt-copynumber-cyto to create the intermediate files. The following code is an example of how to use Cyto2APT:

### Step 2: Generate a specific output

The R package affy2sv can create objects or files compatible with MAD, R-GADA, snpStats, PLINK and PennCNV. This is done using Cyto2Mad or Cyto2SnpMatrix depending on the desired output.

The function Cyto2Mad creates the files compatible with MAD, R-GADA and with PennCNV. The following code shows how to create the files compatible with MAD:

The first argument, cychp.files, indicates where the intermediate files are stored (in this case it takes the value /home/tmp). The second one, output.name, indicates where the files compatible with MAD will be saved (they will be saved into /home/mad). The third argument specifies the output's format (MAD). The last argument, annotation.file is filled with the path to the annotation file (in CSV format), provided by Affymetrix.

To create the files compatible compatible with PennCNV only the value of output.type needs to be changed from mad to penncnv:

The function Cyto2SnpMatrix is in charge of creating a *SnpMatrix Container*, an object compatible with the R package snpStats. An example of how this function is used:

The argument cychp.files (/home/tmp) takes the path where the intermediate files generated with Cyto2APT are stored. annotation.file is filled with the path to the annotation file (in CSV format), provided by Affymetrix. The output.type is set to snpmatrix to generate the *SnpMatrix Container*.

Setting the value of output.type to plink, and adding and filling the argument output.name with a valid directory, Cyto2SnpMatrix creates a file compatible with PLINK:

### Visualization

affy2sv can create a series of plots to help to perform a quality control process on CytoScan populations. The function Cyto2QCView allows to create three type of plots: 1) a plot to see how a single probe was genotyped for all the population 2) a plot, for a single individual, where the intensities of all its probes are shown 3) a plot, for a single individual, that displays the strength and the contrasts of all its probes. The following code shows how Cyto2QCView can be used:

## Results and discussion

To show how affy2sv can be integrated in pipelines developed in R, two different datasets have been analyzed. Figure 1b shows a schema of these two analysis. Dataset A is used to illustrate how to perform a GWAS using CytoScan data. The same data is used to show how to detect genetic mosaicisms. Dataset B is used to describe how to analyze large CNVs and how to genetoype the well-known 8p23.1 inversion.

**Fig. 2:**
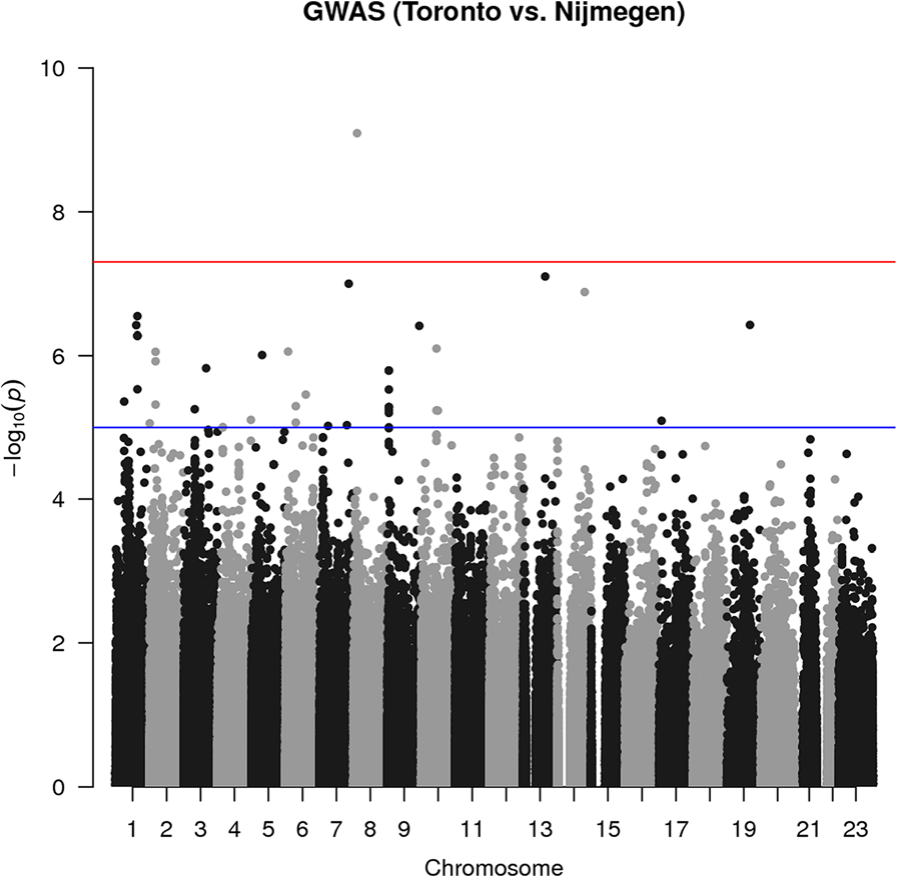
Manhattan plot result of comparing the two populations in Dataset A. Manhattan plot result of the GWAS study comparing general population from Nijmegen versus Toronto (Dataset A), performed with affy2sv and snpStats. It shows the log_10_ of the p-value given to each SNP in chromosome 1 to X

Dataset A includes a set of two populations. 429 subjects corresponding to Toronto general population that comes from The Ontario Population Genomics Platform between the ages of 20 and 79 [48]. Dataset A also includes 198 samples from Nijmegen coming from a full set of 1000 subjects of a previous study [49]. The GWAS consisted in comparing the genotypes between the two populations. We aimed to find the top 10 SNPs that best differentiate the two poplulations. Table 1 shows the results found by using combined efforts of affy2sv and snpStats. Figure 2 shows the Manhattan plot result of this analysis. The complete code to perform this GWAS, including the quality control performed over the SNPs, can be found in supplementary material (Additional file 1). We observe that there is an SNP that passes genome-wide significance level of *p-value < 10* ^*−8*^.

**Table 1:** Results of analyzing Dataset A with aff2sv and snpStats

Name_AFFY_	Name_dbSNP_	CHR	Position	P value	MAF_Nijmegen_	MAF_Toronto_
S-3KHLT	rs2445906	8	87901	7.995824e-10	0.4402516	0.35731132
S-3FEKM	rs12429439	13	516673	7.957754e-08	0.2156250	0.09953162
S-3TIFM	rs62459010	7	688240	1.007604e-07	0.1957831	0.08313253
S-3QSBZ	rs4243640	14	650233	1.317467e-07	0.4108280	0.25768322
S-3XDND	---	1	510137	2.857901e-07	0.1027778	0.03154206
S-4FNLK	rs4239595	19	547436	3.786156e-07	0.2225806	0.10352941
S-4LWCG	rs60081206	1	483264	3.804457e-07	0.1655844	0.06721698
S-4HMDR	rs10868728	9	752489	3.880796e-07	0.4024390	0.25817757
S-3FSKC	rs12402205	1	510072	5.298163e-07	0.1027778	0.03271028
S-3KMMG	---	1	510066	5.298163e-07	0.1027778	0.03271028

Top 10 significant SNPs obtained from the GWAS Toronto vs. Nijmegen (Dataset A) using a complete set of 429 .CEL files from Affymetrix CytoScan HD

**Fig. 3:**
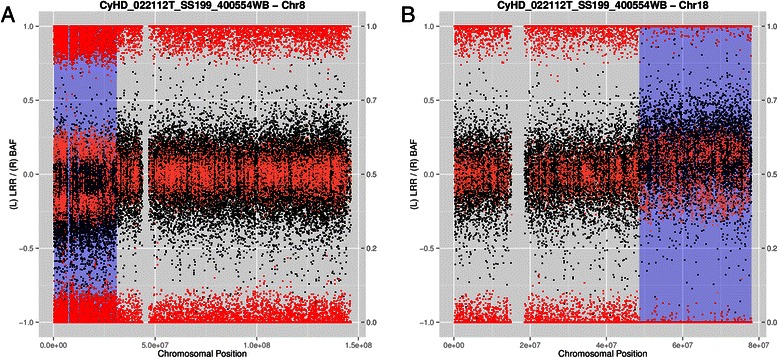
Two of the three mosaic events detected by MAD of Dataset A after being pre-processed by affy2sv. The plots show two mosaic events found by MAD after the pre-process Dataset A with affy2sv Each plot represents the whole chromosome where the mosaic event is located. The black dots show the value of the LRR for each single SNP while the red points show the value of the BAF; placing at the top the ones corresponding to AA allele (with a value close to 1), at the middle the ones corresponding to AB allele (with a value around 0.5) and at the bottom the ones corresponding to BB allele (with a value close to 0). Part **A** shows a 33 Mb mosaic deletion at terminal 8p. Part **B** shows a 25 Mb mosaic duplication at terminal 18q. The presence of both events in the same sample (from Toronto general population) indicated that the individual carries an unbalanced chromosomal translocation (8p; 18q) in a proportion of cells

The mosaicism study in Dataset A was done by using the R package MAD (Additional file 1). Table 2 shows the three unique events found in the entire dataset. Figure 3 shows two of the three events found in the Toronto population. They correspond to a mosaic terminal deletion of 22 Mb at chromosome 8p and a mosaic of 35 Mb gain at terminal of chromosome 18q of the same individual.

**Table 2:** Results of analyzing Dataset A with aff2sv and MAD

IniProbe	EndProbe	LenProbe	CHR	LRR	Bdev	State	Sample	Pop
219677	33114837	279	8	−0.14	0.088	2	CyHD_022112T_SS199_400554WB	T
43717666	78010194	285	18	0.15	0.07	3	CyHD_022112T_SS199_400554WB	T
20520198	107105043	1140	14	0	0.252	1	N_Blood_control99	N

Mosaic events detected by MAD (T = 7, MinSegLen = 100) on the 627 .CEL files from Afymetrix CytoScan HD corresponding to the two general populations of Nijmegen and Toronto in Dataset A. Each column of the table has its own meaning. IniProbe and EndProbe place the mosaic event on the chromosome given by column CHR. The column LenProbe informs of the number of probes in the region detected as a mosaic event, the columns LRR and Bdev are the measures used to detect the mosaic event and to make a previous attempt to classify it. state shows the result of this classification (being 1 = uniparental disomy (UPD), 2 = Deletion, 3 = Duplication, 4 = Trisomy and 5. = loss of heterozygosity (LOH)). sample tells on which sample the mosaic event was found, and the population on which one of both populations, Toronto (T) or Nijmegen (N), the sample belongs to

**Fig. 4:**
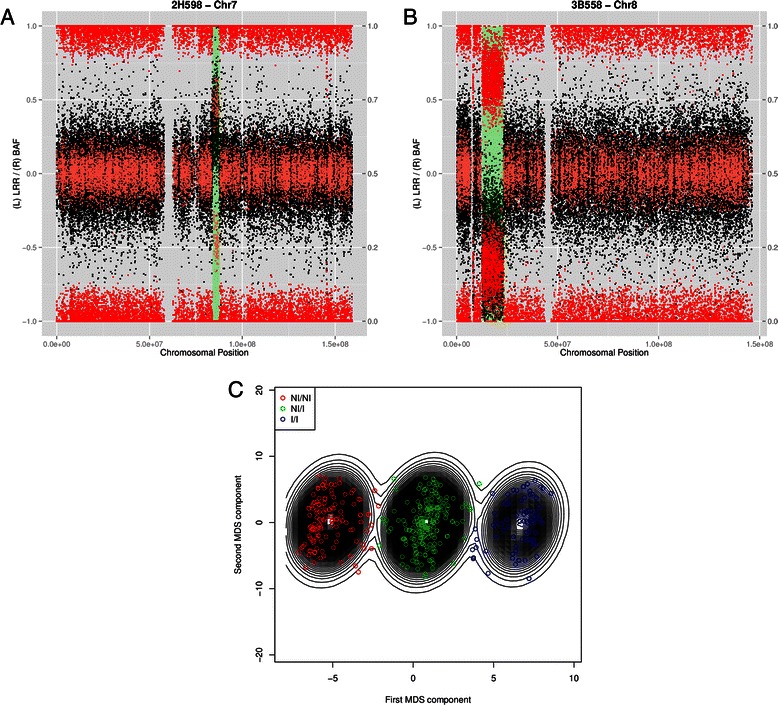
Two CNV events found in the Dataset B population (diagnosed with intellectual disability) with R-GADA and calling of 8p23.1 inversion in the same population with invClust. Part **A** shows a subject from Dataset B having an interstitial gain on chromosome 7q. The black dots show the value of the LRR for each single SNP while the red points show the value of the BAF; placing at the top the ones corresponding to AA allele (with a value close to 1), at the middle the ones corresponding to AB allele (with a value around 0.5) and at the bottom the ones corresponding to BB allele (with a value close to 0). Part **B** shows another subject from the same Dataset B having a interstitial loss on chromosome 8p. Part **C** Shows the calling of the inversion 8p23.1 in the entry dataset. The three groups correspond to each genotype; being the blue points the individuals that contain the inversion in both alleles, the green corresponds to the heterozygous individuals and the red ones are the individuals without the inversion. The plot is obtained after performing a MDS reduction over the population. 2-D density curves indicate the probability of belonging to each genotype being the more closed circles the highest probability

Dataset B includes 315 subjects with intellectual disability from the Biobank of the Galician Foundation of Genomics Medicine (the use of the samples for this purpose was authorized by the Ethical Committee of the institution). For the CNV study the R package R-GADA was used to detect regions with copy gains and losses (Additional file 1). The detected CNVs can be seen in Table 3. Two of these events are represented in Fig. 4a and Fig. 4b. The plots show an interstitial gain in chromosome 7q and an interstitial loss in chromosome 8p in two subjects diagnosed with intellectual disability. Finally, Dataset B is used to genotype 8p23.1 inversion. This was performed using the R package invClust (Additional file 1). The classification of each individual of the population, according to the inversion haplotypes, can be seen in Fig. 4c. Genotype frequencies were: 87 for the allele I/I, 197 to the NI/I and 61 to NI/NI (being I the inverted allele and NI the non-inverted). The inversion appears with a frequency of 46 % in Dataset B population, similar than in general population.

**Table 3:** Results of analyzing Dataset A with aff2sv and R-GADA

IniProbe	EndProbe	LenProbe	MeanAmp	CHR	State	Sample
66690197	71078462	183	0.953	3	−1	1F549
94236184	117023549	447	0.237	X	−1	1J014
52942	15383670	602	0.086	17	−1	1J567
17309881	21217575	121	0.132	22	1	1K397
17309881	21421319	127	0.377	22	−1	2A419
143559	15049329	495	0.890	18	−1	2B595
17309881	21364849	124	0.185	22	1	2D325
65997819	69181942	80	0.049	4	−1	2F584
22759438	32409066	444	0.130	15	−1	2G029
17309881	20192331	109	0.362	22	−1	2G159
83885323	86767689	117	0.265	7	1	2H598
4723882	27966028	709	1.055	5	−1	2L046
143559	11602053	416	0.072	18	−1	2L217
22759438	32409066	444	0.172	15	−1	3A913
12585825	23193309	337	0.837	8	−1	3B558
218476969	249191732	1144	0.070	1	−1	3C103
144131822	159100528	652	0.971	7	−1	8D582
134476	18433821	591	0.466	20	1	8D582
15529890	21862551	208	0.367	8	1	P609

CNVs detected by R-GADA (T = 7, MinSegLen = 100) on the 315 .CEL files from Afymetrix CytoScan HD corresponding to the population diagnosed with intellectual disability (ID) in Dataset B. The table is the result of the exportation of the object created by R-GADA. The columns IniProbe, EndProbe, chromosome and LenProbe tells us how many probes are contained din the region detected as CNV event, the column sample shows the sample's name containing the CNV. The value given by MeanAmp is used to try to classify the event (in gain or loss), the result of this classification is seen in State (1: gain; −1: loss)

**Fig. 5:**
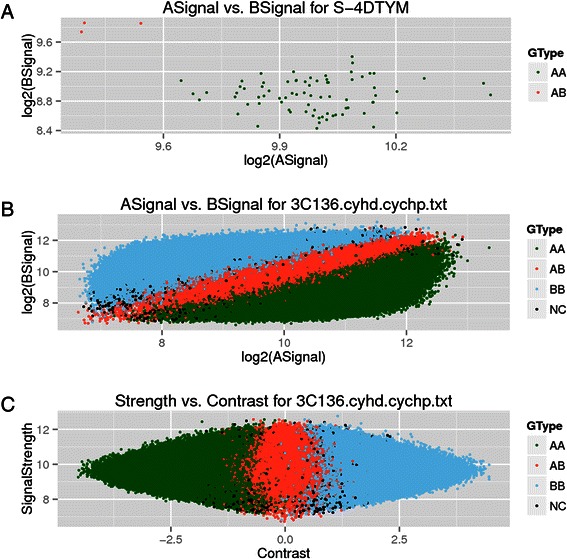
The three type of plots affy2sv can draw on CytoScan samples to perform a visual QC. Plot **A** shows the log_2_ of the intensities of both alleles for a single SNP across all the population. In the case, a random probe (A-4DTYM) was selected and drawn across the population diagnosed with intellectual disability (Dataset B). Plot **B** shows the values corresponding to the log_2_ of the intensity of both alleles for all the probes in a random subject (3C136, from Dataset B). Plot **C** draws the strength and the contrast of all the probes for a random individual (3C136), being the strength log(A + B) and contrast (A-B)/(A + B)

The R package affy2sv includes a function to perform a simple but visual quality control on CytoScan samples. A plot where a single SNP is displayed for all samples can be created (Fig. 5a). Another plot for allele intensities (Fig. 5b) or a plot that shows the strength versus the contrast of each probe (Fig. 5c). See Additional file 1.

## Conclusion

affy2sv is an R package to pre-process raw .CEL files from Affymetrix CytoScan HD and 750k arrays (also the old SNP arrays called Genome-Wide SNP 5.0/6.0 and Axiom). The package can be used to create a wide range of output files and object compatibles with other R packages, like snpStats or MAD, and external software, like PLINK and PennCNV, used in genetic structural variants studies.

### Availability & requirements

- Package's name: affy2sv
- Package's state: affy2sv 1.0.12 with APT 1.16.1
- Package's web page: affy2sv is available at Bioinformatic Research Group in Epidemiology (BRGE - CREAL) software page http://www.creal.cat/brge.htm. Also at its own page on bitbucket https://bitbucket.org/brge/affy2sv.
- Package's manual: The package comes with its standard R documentation. A web page manual is available at the packages own page on bitbucket https://bitbucket.org/brge/affy2sv/wiki.
- Package's requirements:
  - ○ operating systems: Multiplatform (Windows, GNU/Linux and MAC OS)
  - ○ r dependence: R (> = 3.0.0), snpStats, crlmm, oligo, oligoClasses, VanillaICE, SNPchip, genomewidesnp6Crlmm, genomewidesnp5Crlmm, ff, pd.genomewidesnp.6, pd.genomewidesnp.5, stringr, biomaRt, ggplot2, gtable, grid, data.table, Biobase, parallel, methods
  - ○ external dependences: python 2.7, numpy (> = 1.7), pandas
- Programming language: R, Python and C/C++
- License: GPL-2
- Any restrictions to use by non-academics: No restrictions to use affy2sv, check the license for APT at its own web page.

## Additional file

Below is the link to the electronic supplementary material.

Additional file 1: **It includes a plot for mosaic events detected in Dataset A and for the CNV events detected in Dataset B.** It also includes the full code used in both analysis of both Dataset A and Dataset B.
