# Supplementary material for: affy2sv: an R package to pre-process Affymetrix CytoScan HD and 750K arrays for SNP, CNV, inversion and mosaicism calling
Source: BMC Bioinformatics. 2015 May 20;16:167. doi: 10.1186/s12859-015-0608-y (PMC4438530; doi:10.1186/s12859-015-0608-y)
Supplement: Supplementary file 1 — It includes a plot for mosaic events detected in Dataset A and for the CNV events detected in Dataset B. It also includes the full code used in both analysis of both Dataset A and Dataset B. [file 12859_2015_608_MOESM1_ESM.pdf]

Supplementary Material

`affy2sv`: An R package to preprocess Affymetrix CytoScan HD and 750K arrays in SNP, CNV, inversion and mosaicism calling

Contents

Mosaic Events **2**

CNV Events **4**

R code **13**

R code used to perform the GWAS 14

R code used to detect mosaicism 17

R code used to detect CNVs 18

R code used for 8p23.1 inversion calling 19

R code used for QC plots on CytoScan samples 20

Content of `roi.txt` 21

Mosaic Events

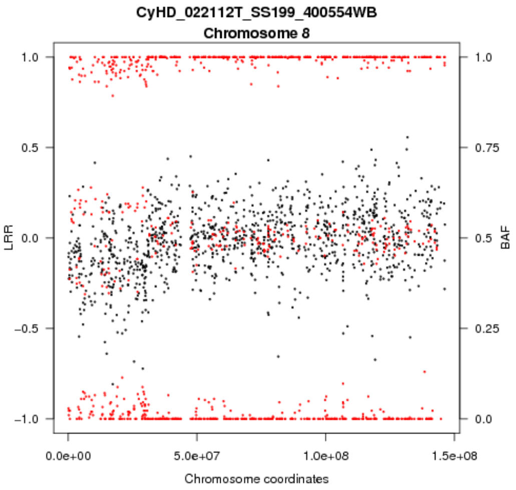

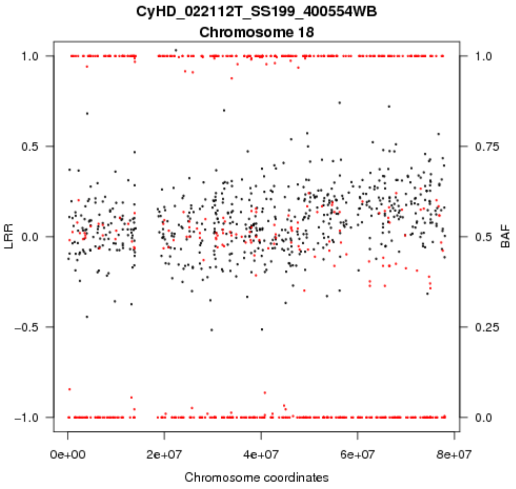

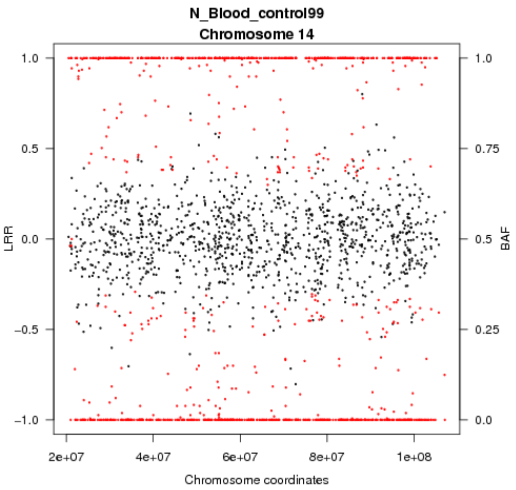
N_Blood_control99
Chromosome 14
LRR
BAF
Chromosome coordinates
1.0
0.5
0.0
−0.5
−1.0
0.75
0.25
2e+07
4e+07
6e+07
8e+07
1e+08

CNV Events

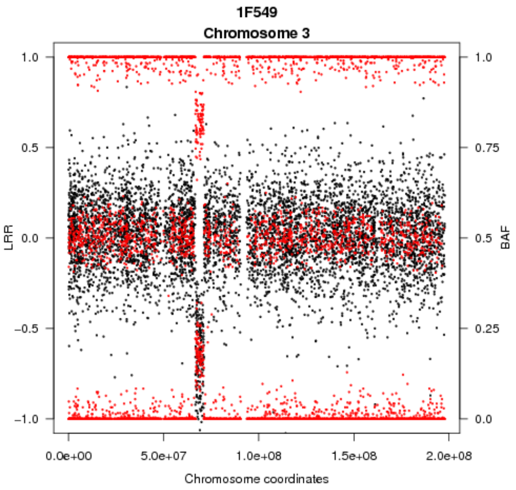
1F549
Chromosome 3
LRR
BAF
Chromosome coordinates
1.0
0.5
0.0
−0.5
−1.0
0.75
0.25
0.0e+00
5.0e+07
1.0e+08
1.5e+08
2.0e+08

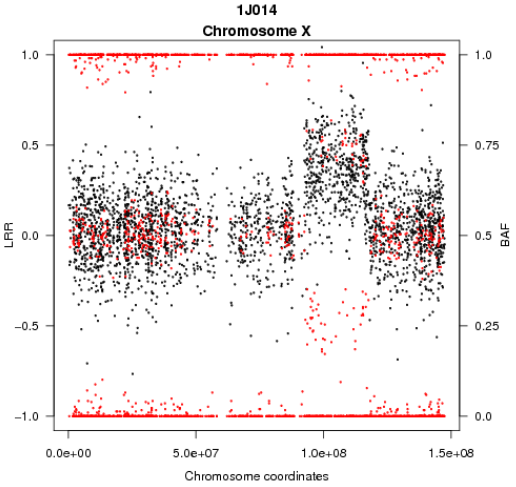
1J014
Chromosome X
LRR
BAF
Chromosome coordinates
1.0
0.5
0.0
−0.5
−1.0
0.75
0.25
0.0e+00
5.0e+07
1.0e+08
1.5e+08

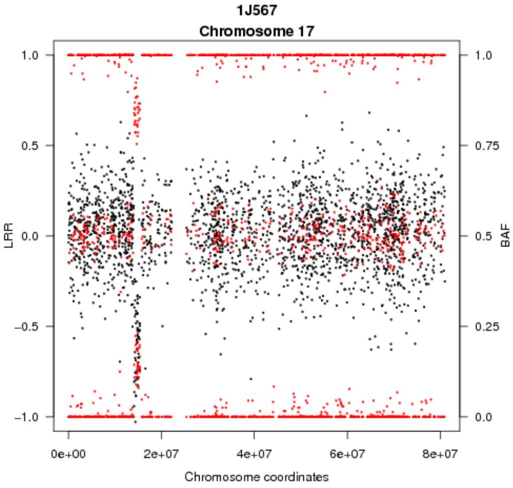
1J567
Chromosome 17
LRR
BAF
1.0
0.5
0.0
−0.5
−1.0
1.0
0.75
0.5
0.25
0.0
0e+00
2e+07
4e+07
6e+07
8e+07
Chromosome coordinates

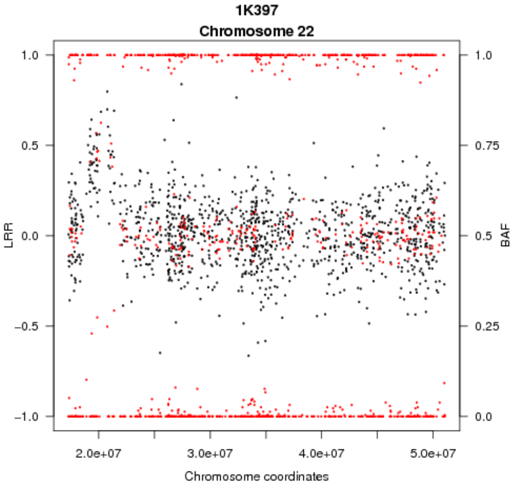
1K397
Chromosome 22
LRR
BAF
1.0
0.5
0.0
−0.5
−1.0
1.0
0.75
0.5
0.25
0.0
2.0e+07
3.0e+07
4.0e+07
5.0e+07
Chromosome coordinates

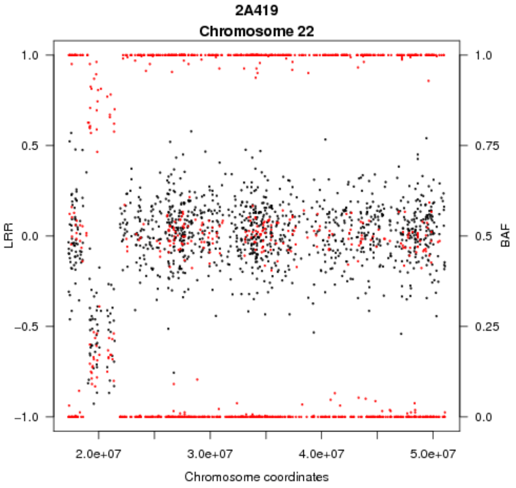
2A419
Chromosome 22
LRR
BAF
1.0
0.5
0.0
−0.5
−1.0
1.0
0.75
0.5
0.25
0.0
2.0e+07
3.0e+07
4.0e+07
5.0e+07
Chromosome coordinates

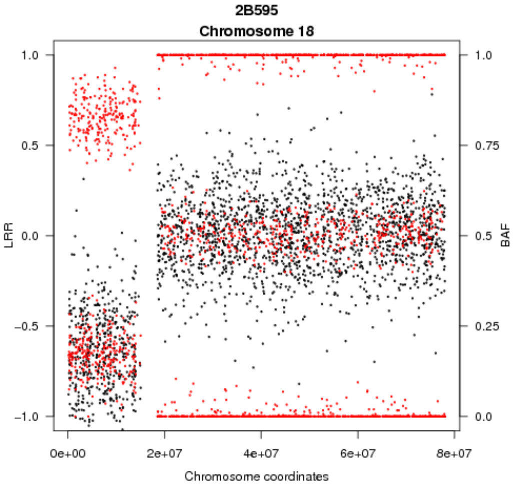
2B595
Chromosome 18
LRR
BAF
1.0
0.5
0.0
−0.5
−1.0
1.0
0.75
0.5
0.25
0.0
0e+00
2e+07
4e+07
6e+07
8e+07
Chromosome coordinates

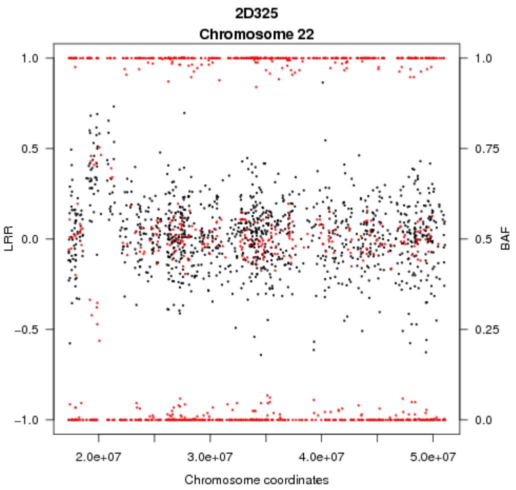
2D325
Chromosome 22
LRR
BAF
1.0
0.5
0.0
−0.5
−1.0
1.0
0.75
0.5
0.25
0.0
2.0e+07
3.0e+07
4.0e+07
5.0e+07
Chromosome coordinates

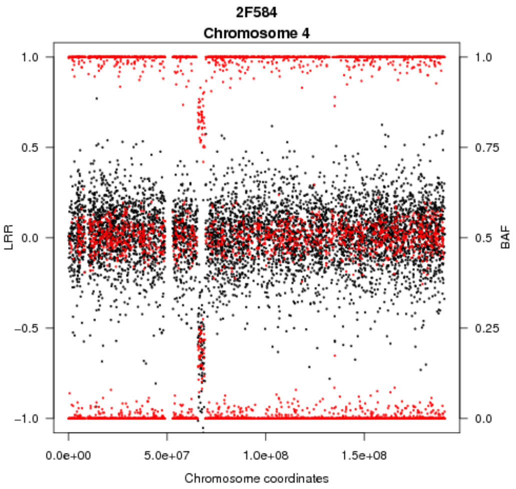
2F584
Chromosome 4
LRR
BAF
1.0
0.5
0.0
−0.5
−1.0
1.0
0.75
0.5
0.25
0.0
0.0e+00
5.0e+07
1.0e+08
1.5e+08
Chromosome coordinates

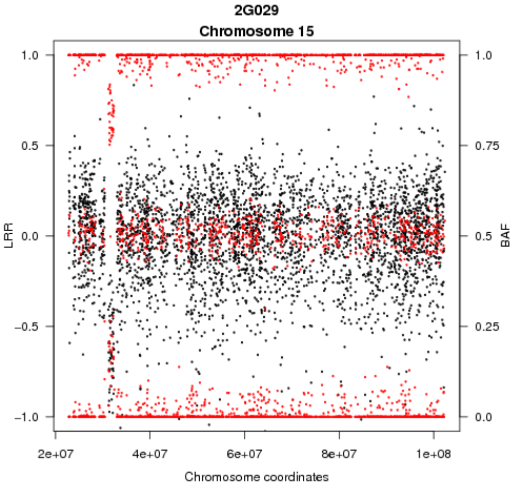
2G029
Chromosome 15
LRR
BAF
Chromosome coordinates
2e+07
4e+07
6e+07
8e+07
1e+08

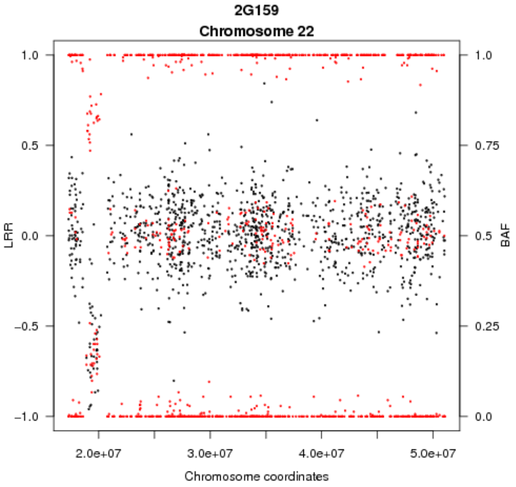
2G159
Chromosome 22
LRR
BAF
Chromosome coordinates
2.0e+07
3.0e+07
4.0e+07
5.0e+07

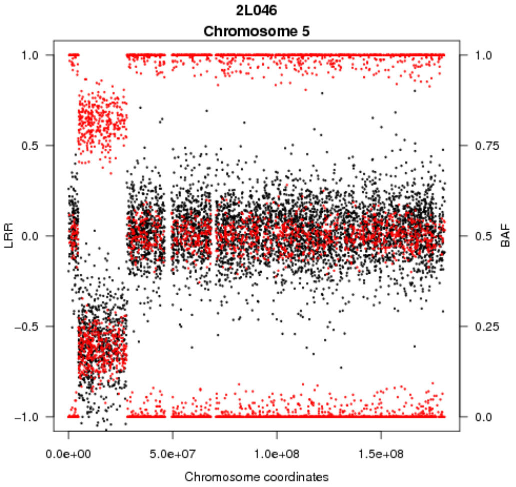
2L046
Chromosome 5
LRR
BAF
Chromosome coordinates
0.0e+00
5.0e+07
1.0e+08
1.5e+08
1.0
0.5
0.0
−0.5
−1.0
1.0
0.75
0.5
0.25
0.0

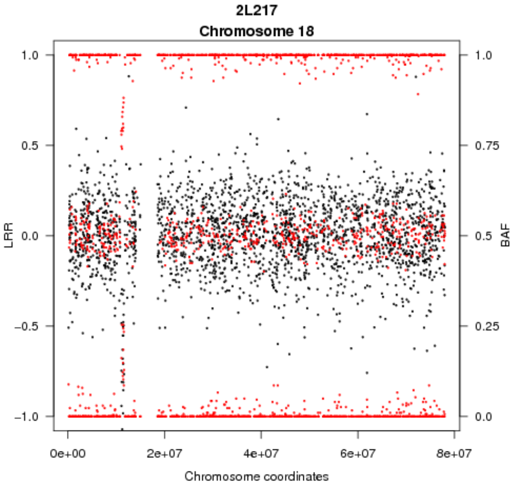
2L217
Chromosome 18
LRR
BAF
Chromosome coordinates
0e+00
2e+07
4e+07
6e+07
8e+07
1.0
0.5
0.0
−0.5
−1.0
1.0
0.75
0.5
0.25
0.0

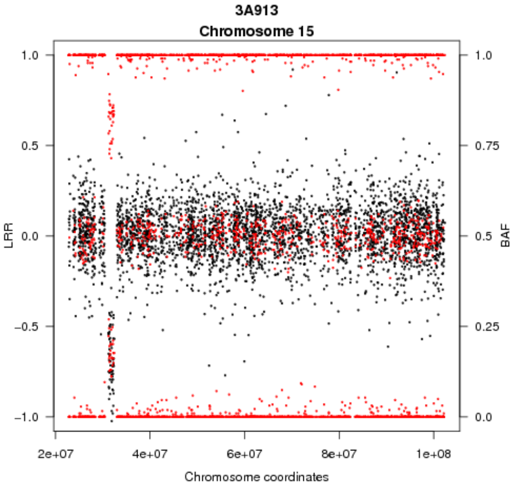
3A913
Chromosome 15
LRR
BAF
1.0
0.5
0.0
−0.5
−1.0
0.75
0.25
2e+07
4e+07
6e+07
8e+07
1e+08
Chromosome coordinates

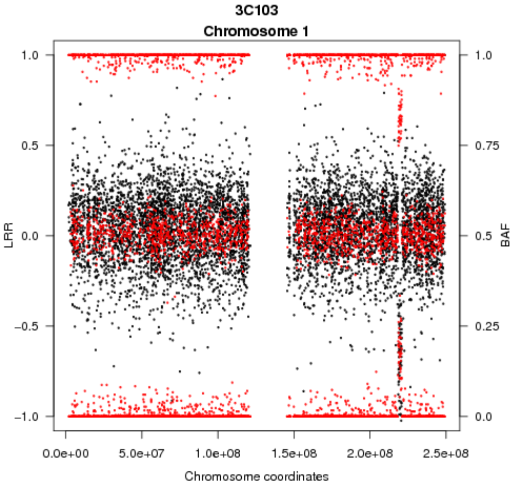
3C103
Chromosome 1
LRR
BAF
1.0
0.5
0.0
−0.5
−1.0
0.75
0.25
0.0e+00
5.0e+07
1.0e+08
1.5e+08
2.0e+08
2.5e+08
Chromosome coordinates

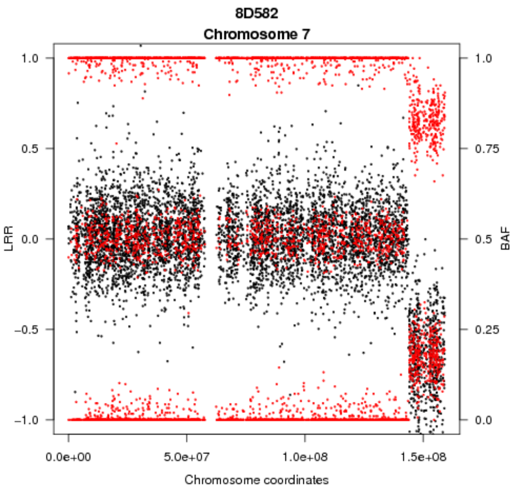
8D582
Chromosome 7
LRR
BAF
Chromosome coordinates
1.0
0.5
0.0
−0.5
−1.0
0.75
0.25
0.0e+00
5.0e+07
1.0e+08
1.5e+08

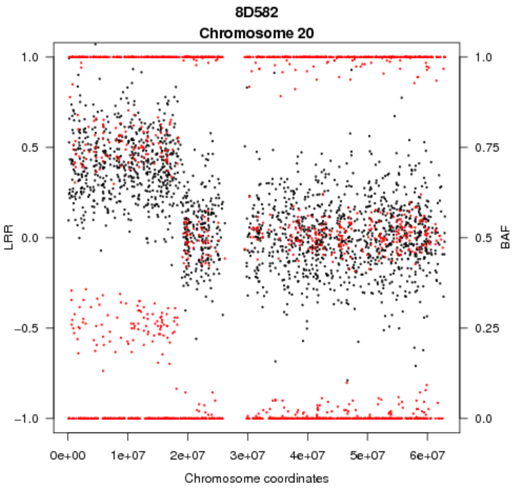
8D582
Chromosome 20
LRR
BAF
Chromosome coordinates
1.0
0.5
0.0
−0.5
−1.0
0.75
0.25
0e+00
1e+07
2e+07
3e+07
4e+07
5e+07
6e+07

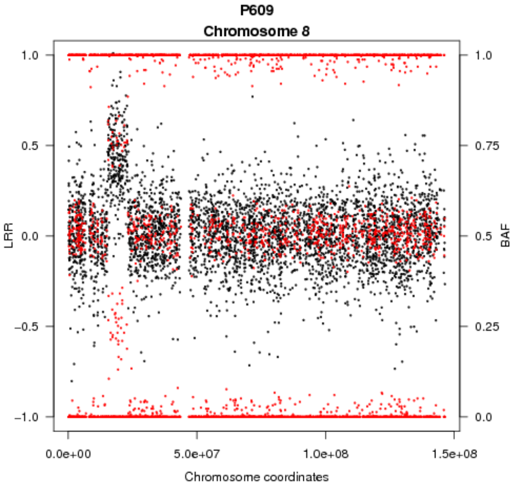

P609
Chromosome 8
LRR
BAF
Chromosome coordinates
1.0
0.5
0.0
−0.5
−1.0
0.75
0.25
0.0e+00
5.0e+07
1.0e+08
1.5e+08

R code

(Sup. Code 1) R code for 'standard' and 'custom' call of `Cyto2APT`

The following is the code to execute a "standard" run of the `Cyto2APT`:

```
aptParam <- APTparam(
    type="cytoscan",
    level="standard",
    cel.list="~/cydata",
    output.path="~/tmp",
    analysis.path="/home/chernandez/lib/cyhd/",
    cdf="CytoScanHD_Array.cdf",
    chrX="CytoScanHD_Array.chrXprobes",
    chrY="CytoScanHD_Array.chrYprobes",
    qca="CytoScanHD_Array.r1.qca",
    qcc="CytoScanHD_Array.r1.qcc",
    snp="CytoScanHD_Array.snplist.txt",
    annot.db="CytoScanHD_Array.na32.3.annot.db",
    refmodel="CytoScanHD_Array.na32.3.v1.REF_MODEL"
)
Cyto2APT(aptParam)
```

The following is the code to execute the same run but with the "custom" mode of `APTparam`:

```
aptArgs <- paste0(   " -v 4  --cyto2 false --doDualNormalization true",
          " --keep-intermediate-data false",
          " --run-geno-qc true",
          " --snp-qc-use-contrast true",
          " --force true",
          " --adapter-type-normalization false",
          " --text-output true",
          " --cnchp-output false",
          " --cychp-output true",
          " --set-analysis-name cyhd",
          " --analysis loh-segment",
          " --analysis cn-neutral-loh",
          " --male-gender-ratio-cutoff 1.5",
          " --female-gender-ratio-cutoff 0.9",
          " --xx-cutoff 0.61",
          " --xx-cutoff-high 0.95",
          " --y-cutoff 0.58",
          " --local-gc-background-intensity-adjustment-method none",
          " --image-correction-intensity-adjustment-method none",
          " --wave-correction-log2ratio-adjustment-method wave"
          "-correction-log2ratio-adjustment-method.bandwidth=101.",
          "bin-count=25.wave-count=6.wave-smooth=true",
          " --cn-calibrate-parameters calibrated-log2ratios.alpha-cn-",
          "calibrate=0.564278.alpha-X-cn-calibrate=0.619453.alpha-Y-cn-",
          "calibrate=0.494620.beta-cn-calibrate=1.beta-X-cn-calibrate=1.",
          "beta-Y-cn-calibrate=1",
          " --analysis genotype --analysis log2-ratio.gc-correction=false.",
          "median-autosome-median-normalization=true.median-smooth-marker-count=5",
          " --log2ratio-adjustment-method log2ratio-adjustment-method-high-pass-filter.use=true",
          " --analysis allelic-difference-CytoScan.outlier-trim=3.0.step=20.window=100"
          ".point-count=128.bandwidth=0.25.cutoff=0.05.clean-threshold=0.35.symmetry=true ",
          " --analysis kernel-smooth.sigma_span=50",
          " --analysis cn-cyto2.",
          "hmmCN_state=\\'0,1,2,3,4\\'.",
          "hmmCN_mu=\\'-2,-0.45,0,0.3,0.51\\'.",
```

```
        "hmmCN_sigma=\\'0.35,0.35,0.25,0.25,0.25\\'.",
        "hmmCN_state-X=\\'0,1,2,3,4\\'.",
        "hmmCN_mu-X=\\'-2,-0.47,0,0.33,0.53\\'.",
        "hmmCN_sigma-X=\\'0.35,0.35,0.25,0.25,0.25\\'.",
        "hmmCN_state-Y=\\'0,1,2,3,4\\'.",
        "hmmCN_mu-Y=\\'-2,-0.45,0,0.3,0.51\\'.",
        "hmmCN_sigma-Y=\\'0.35,0.35,0.25,0.25,0.25\\'.",
        "diagonal-weight-Y=0.995.",
        "mapd-weight-Y=0.",
        "min-segment-size-Y=5.",
        "hmm-confidence-weight-Y=0.6.",
        "diagonal-weight=0.995.",
        "mapd-weight=0.",
        "min-segment-size=5.",
        "hmm-confidence-weight=0.6.",
        "diagonal-weight-X=0.995.",
        "mapd-weight-X=0.",
        "min-segment-size-X=5.",
        "hmm-confidence-weight-X=0.6.",
        "shrink=true ",
        " --analysis cn-cyto2-gender.cutoff=0.5 --analysis cn-segment",
        " --analysis lohCytoScan.lohCS_errorrate=0.05.lohCS_beta=0.001.lohCS_alpha=0.01.lohCS_separation=1000000.",
        "lohCS_nMinMarkers=10.lohCS_NoCallThreshold=0.05.lohCS_minGenomicSpan=1000000",
        " --qca-file /home/chernandez/lib/cyhd/CytoScanHD_Array.r1.qca",
        " --qcc-file /home/chernandez/lib/cyhd/CytoScanHD_Array.r1.qcc",
        " --snp-qc-snp-list /home/chernandez/lib/cyhd/CytoScanHD_Array.snplist.txt",
        " --cdf-file /home/chernandez/lib/cyhd/CytoScanHD_Array.cdf",
        " --chrX-probes /home/chernandez/lib/cyhd/CytoScanHD_Array.chrXprobes",
        " --chrY-probes /home/chernandez/lib/cyhd/CytoScanHD_Array.chrYprobes",
        " --annotation-file /home/chernandez/lib/cyhd/CytoScanHD_Array.na32.3.annot.db",
        " --reference-input /home/chernandez/lib/cyhd/CytoScanHD_Array.na32.3.v1.REF_MODEL",
        " --cel-files cydata",
        " --out-dir tmp"
)

aptParam <- APTparam(
    type="cytoscan",
    level="custom",
    param=aptArgs
)
Cyto2APT(aptParam)
```

(Sup. Cod. 2) R code used to perform the GWAS

We must notice that genotype calling performed using the `Cyto2APT`, and for extension by APT, produced a certain number of bad quality SNPs. Therefore some quality control filters were used to discard these low quality SNPs (default settings from `apt-copynumber.cyto`), keeping a total of 550170 genotyped and good quality SNPs on all the 429 samples, Individuals with call rate <98% were removed. SNPs with call rate <95% and MAF <5% were also discarded from the analysis.

```
# 1. LOAD LIBRARY
# ############################################################################ #
library( snpStats )

# 2. LOAD DATA
# ############################################################################ #
ind.info <- read.table(
```

```
    file   =  "/DATA/claind.tsv",
    header = TRUE,
    sep    = "\t"
)

load( "smc_tornonto_nijmegen.rda" )

# 3. SAME ORDER IN ALL OBJECTS
# ########################################################################## #
order.sam            <- rownames( smc$genotype )
rownames( ind.info ) <- ind.info$Sample
ind.info             <- ind.info[ order.sam, ]

# 4. FILTER SAMPLES
# ########################################################################## #
infoInd <- row.summary( smc$genotype )
useInd  <- infoInd$Heterozygosity > 0.2 & infoInd$Call.rate > 0.98

geno    <- smc$genotype[ useInd, ]


# 5. ASSOCIATION
# ########################################################################## #
cc    <- ind.info$CC
tests <- single.snp.tests( cc[ useInd ],
    data     = ind.info[ useInd, ],
    snp.data = geno
)

# 6. FILTER SNPs
# ########################################################################## #
infoSnp <- col.summary( geno )
infoSnp$pvalHWE <- 1 - pnorm( infoSnp$z.HWE )
useSnp  <- !is.na( infoSnp$MAF ) & infoSnp$MAF > 0.05 &
    infoSnp$pvalHWE > 0.001 & infoSnp$Call.rate > 0.95

tests.f <- tests[ useSnp, ]


# 7. GENRATE TABLE
# ########################################################################## #
pval  <- p.value( tests.f, df=1 )
ord   <- order( pval )
top10 <- ord[ 1:10 ]
```

```
names.affy <- tests.f@snp.names[ top10 ]
position   <- smc$map[ names.affy, "position" ]
chromosome <- smc$map[ names.affy, "chromosome" ]
names      <- smc$map[ names.affy, "snp.name" ]

top10      <- data.frame(
    Name    = names,
    Chr     = chromosome,
    Pos     = position,
    PVal    = pval[ top10 ],
    MAF     = infoSnp[ names.affy, "MAF" ],
    PValHWE = infoSnp[ names.affy, "pvalHWE" ]
)

# 8. SAVE TABLE
# ############################################################################ #
write.table(top10,
    col.names = TRUE,
    row.names = FALSE,
    quote     = FALSE,
    sep       = "\t",
    file      = "top10snps.tsv"
)

# 9. SAVE ALL VALUES
# ############################################################################ #
names.affy <- tests.f@snp.names
pVal <- data.frame(
    Name    = names.affy,
    Chr     = smc$map[ names.affy, "chromosome" ],
    Pos     = smc$map[ names.affy, "position" ],
    PVal    = p.value( tests.f, df=1 )
)

write.table(pVal,
    col.names = TRUE,
    row.names = FALSE,
    quote     = FALSE,
    sep       = "\t",
    file      = "pValSnps.tsv"
)
```

(Sup. Cod. 3) R code used to detect mosaicism

```
# 1. LOAD LIBRARY
# ############################################################################## #
library( gada )
library( multicore )

# 2. SET SETINGS
# ############################################################################## #
path <- "/DATA/" # where the folder 'rawData' with the MAD-files is placed
options( cores = 8 ) # number of cores used by MAD (gada)

# 3. SETUP
# ############################################################################## #
# The header of a MAD-file (CytoScan) contains 6 labels:
#  1. Name          4. Log.R.Ratio
#  2. Chrm          5. GType
#  3. Position      6. B.Allele.Freq

object <- setupParGADA.B.deviation(
    folder    = path,
    NumCols   = 6,
    log2ratio = 4,
    GenoCol   = 5,
    BAFcol    = 6
)

# OPTIONAL:
# save( object, file="setupMAD.RData" )

# 4. SBL procedure
# ############################################################################## #
parSBL( object,
    estim.sigma2 = TRUE,
    aAlpha       = 0.8
)

# 5. Backward Elimination
# ############################################################################## #
parBE.B.deviation( object,
    T         = 7,
    MinSegLen = 100
)
```

```
# OPTIONAL
# exportSegments2File( object, file="MAD_T_7_Min_100.txt" )
```

(Sup. Cod. 4) R code used to detect CNVs

```
# 1. LOAD LIBRARY
# ############################################################################ #
library( gada )
library( multicore )

# 2. SET SETINGS
# ############################################################################ #
path <- "/DATA/" # where the folder 'rawData' with the MAD-files is placed
options( cores = 8 ) # number of cores used by GADA

# 3. SETUP
# ############################################################################ #
# The header of a MAD-file (CytoScan) contains 6 labels:
#  1. Name          4. Log.R.Ratio
#  2. Chrm          5. GType
#  3. Position      6. B.Allele.Freq

object <- setupParGADA.B.deviation(
    folder    = path,
    NumCols   = 6,
    log2ratio = 4,
    GenoCol   = 5,
    BAFcol    = 6
)

# OPTIONAL:
# save( object, file="setupGADA.RData" )

# 4. SBL procedure
# ############################################################################ #
parSBL( object,
    estim.sigma2 = TRUE,
    aAlpha       = 0.8
)

# 5. Backward Elimination
```

```
# ########################################################################## #
parBE( object,
    T         = 7,
    MinSegLen = 100
)

# OPTIONAL
# exportSegments2File( object, file="GADA_T_7_Min_100.txt" )
```

(Sup. Cod. 5) R code used for 8p23.1 inversion calling

```
# 1. LOAD LIBRARY
# ########################################################################## #
library( affy2sv )
library( invClust )

# 2. LOAD DATA
# ########################################################################## #
load( "smc_asd.rda" )
load( "smc_id.rda" )

# 3. CONVERT ANNOTATION
# ########################################################################## #
# Converting the Affymetrix's SNP-annotation to standard HG19

smc_asd <- update_map( smc_asd )
smc_id  <- update_map( smc_id )

# 4. PREPARE THE ANNOTATION FOR invClust
# ########################################################################## #
annot_asd <- smc_asd$map[ , c( "snp.name", "chromosome", "position" ) ]
annot_id  <- smc_id$map[ , c( "snp.name", "chromosome", "position" ) ]

names( annot_asd ) <- c( "rs", "chromosome", "position" )
names( annot_id ) <- c( "rs", "chromosome", "position" )

# 5. PREPARE THE GENOTYPE MATRIX FOR invClust
# ########################################################################## #
annot_asd <- annot_asd[ annot_asd$chromosome != "Y", ]
annot_id  <- annot_id[ annot_asd$chromosome != "Y", ]

geno_asd <- smc_asd$genotype[ , rownames( annot_asd ) ]
```

```
geno_id <- smc_id$genotype[ , rownames( annot_id ) ]

# 6. RUNNING invClust
# ############################################################################# #
inv <- invClust(
    roi = "roi.txt",
    wh  = 1,
    geno,
    annot,
    dim = 2,
    tol = 1e-5
)

# OPTIONAL:
# inv

# OPTIONAL:
# plot( inv )

# OPTIONAL:
# table( invGenotypes( inv ) )
```

(Sup. Cod. 6) R code used for QC plots on CytoScan samples

```
# 1. LOAD LIBRARY
# ############################################################################# #
library( affy2sv )


# 2. PLOT SNP FOR ALL SAMPLES
# ############################################################################# #
config <- CytoQCView(path="APT", visualization="snp",
individual="S-4DTYM")
snpPlot <- plot(config)

# 3. SC PLOT - SINGLE SAMPLES
# ############################################################################# #
config <- CytoQCView(path="APT", visualization="sc",
individual="3C136.cyhd.cychp.txt")
scPlot <- plot(config)

# 4. INTENSITY PLOT - SINGLE SAMPLE
```

```
# ############################################################################ #
config <- CytoQCView(path="APT", visualization="int",
individual="3C136.cyhd.cychp.txt")
intPlot <- plot(config)
```

Content of roi.txt

The content of the file roi.txt determines the region of interest where the inversion can be found (hg19):

```
chr  LBP      RBP       reg       method  tagSNPs
8    7897515  11787032  7.9-11.8  1       NA
```
